# Bilateral facet effusion is a risk factor for segmental instability with cervical injury without vertebral fracture

**DOI:** 10.1038/s41598-021-91981-y

**Published:** 2021-06-15

**Authors:** Shinji Tanishima, Tokumitsu Mihara, Shinya Ogawa, Chikako Takeda, Satoshi Fujiwara, Hideki Nagashima

**Affiliations:** 1 Division of Orthopedic Surgery, Department of Sensory of Motor Organs, School of Medicine, Faculty of Medicine, Tottori University, Yonago, Tottori 683-8504 Japan; 2 Department of Orthopedic Surgery, Japanese Red Cross Masuda Hospital, Masuda, Shimane 698-8501 Japan; 3 Department of Orthopedic Surgery, Sanin Rosai Hospital, Yonago, Tottori 683-0002 Japan

**Keywords:** Diseases, Health care, Medical research, Risk factors, Signs and symptoms

## Abstract

Magnetic resonance imaging (MRI) is effective in identifying cervical spine injury after trauma. However, cervical instability without major bone injury or dislocation is challenging to assess. Hence, the current study aimed to investigate and compare the MRI and radiography findings of segmental instability in patients with cervical spine injury. We investigated 34 participants with cervical spine injury without vertebral fracture. Based on the radiography findings, the participants were categorized into two: group A with segmental instability (n = 11) and group B without segmental instability (n = 23). Both groups were compared in terms of the presence of segmental instability on radiography and MRI. Anterior longitudinal ligament (ALL) injury, disc injury, and bilateral facet effusion were observed in 6/11, 5/11, and 7/11 patients in group A and in 5/23, 2/23 and 7/23 patients in group B, respectively. The results showed significant differences (p < 0.05). Moreover, 2 and 10 of 11 patients in group A and 16 and 7 of 23 patients in group B presented with hemi lateral facet effusion and paravertebral muscle injury, respectively. However, the results did not significantly differ. According to a logistic regression analysis, bilateral facet effusion after trauma was associated with cervical segmental instability (odd ratio: 10.6, 95% confidence interval: 1.31–84.7). Facet joint effusion might be caused by capsule injury during trauma. Most participants with segmental instability had ALL, disc, and flavum injury and bilateral facet effusion. Therefore, we need to consider bilateral facet effusion with other soft tissue damage of the cervical spine as an association factor to show the instability.

## Introduction

Cervical spine injury without major vertebral fractures is common among middle-aged individuals^1^. These cervical injury include intervertebral disc injury or damage to soft tissues, such as the anterior longitudinal ligament (ALL) and yellow ligament.

These injuries occurs might have possibility cervical segmental instability. This ultimately causes spinal cord injury, which requires emergency fixation^2–4^.

As the latter is not associated with abnormalities found on cervical radiography, instability may be overlooked during the initial treatment.

After trauma, swelling of soft tissues located anterior to the cervical spine or abnormality in spinal alignment on cervical radiography indicates cervical spine injury. However, the exact level of injury is challenging to determine. Meanwhile, soft tissue damage on MRI is an indicator of spinal instability^2^. Among the different types of soft tissue damage, edema of the ligaments and paravertebral muscles may be considered significant. However, only few studies have compared the extent of soft tissue damage in actual cervical segmental instability. Maeda et al. showed that ALL and intervertebral disc injuries contribute to instability in hyperextension injury, and these conditions are correlated with the severity of spinal cord injury^3^.

In case of low back pain, facet joint effusion indicates instability and it is an important MRI finding that should be considered before performing fixation^4^. However, the condition is seldom used as an indicator of instability in patients with cervical spine injury. In cases of reduced cervical dislocation, assuming that traumatic facet joint damage has occurred, it can be used as an indicator of instability.

This study aimed to compare the MRI and dynamic radiography findings of cervical spine trauma and to investigate the relationship between facet joint effusion of the cervical spine and cervical segmental instability in trauma cases. Moreover, the association between intervertebral disc and ligament injuries and spinal instability on dynamic radiography of the cervical spine was examined.

## Materials and methods

This was a retrospective study. Patients who underwent dynamic radiography for cervical spine injury without evident vertebral fracture from January 2014 to December 2017 were enrolled. The American Spinal Injury Association Impairment Scale was used to assess for paralysis. The criteria for dynamic radiography of the cervical spine were as follows: no evident cervical dislocation and major bony injury. We regarded a small avulsion fracture of the vertebral body, spinous process fracture, or bone bruise in the vertebral body without noticeable vertebral collapse was considered as a minor bony injury according to previous report^5^ and enrolled these cases inclusion criteria in this study. Meanwhile, the exclusion criteria were vertebral fracture, evident cervical dislocation, spinal infection, and spinal tumor. Radiography was performed by an orthopedic surgeon in collaboration with an emergency physician at the time of or within a few days after injury. Dynamic radiography, with passive anteflexing and retroflexing, was conducted by a physician on the day of injury. Then, it was performed if the patient could tolerate the pain and was discontinued after any complaint of exacerbated neck pain or neurological symptoms in any of the four limbs.

We performed dynamic cervical radiography using a gentle force to prevent neck pain.

We facilitated flexion and extension movements until the identification of abnormal local kyphosis and slippage of the injured segment at the cervical spine. If patients did not present with abnormal symptoms, the same movement was facilitated as tolerated by the patient.

MRI was performed after ruling out contraindications, which include the use of pacemaker and clipping due to subarachnoid hemorrhage.

Assessment was performed according to the report of White et al^6^. Slippage of > 3.5 mm and segmental mobility of > 11° were considered as indicators of instability. Two experienced spine surgeons performed radiography.

**Figure 1:**
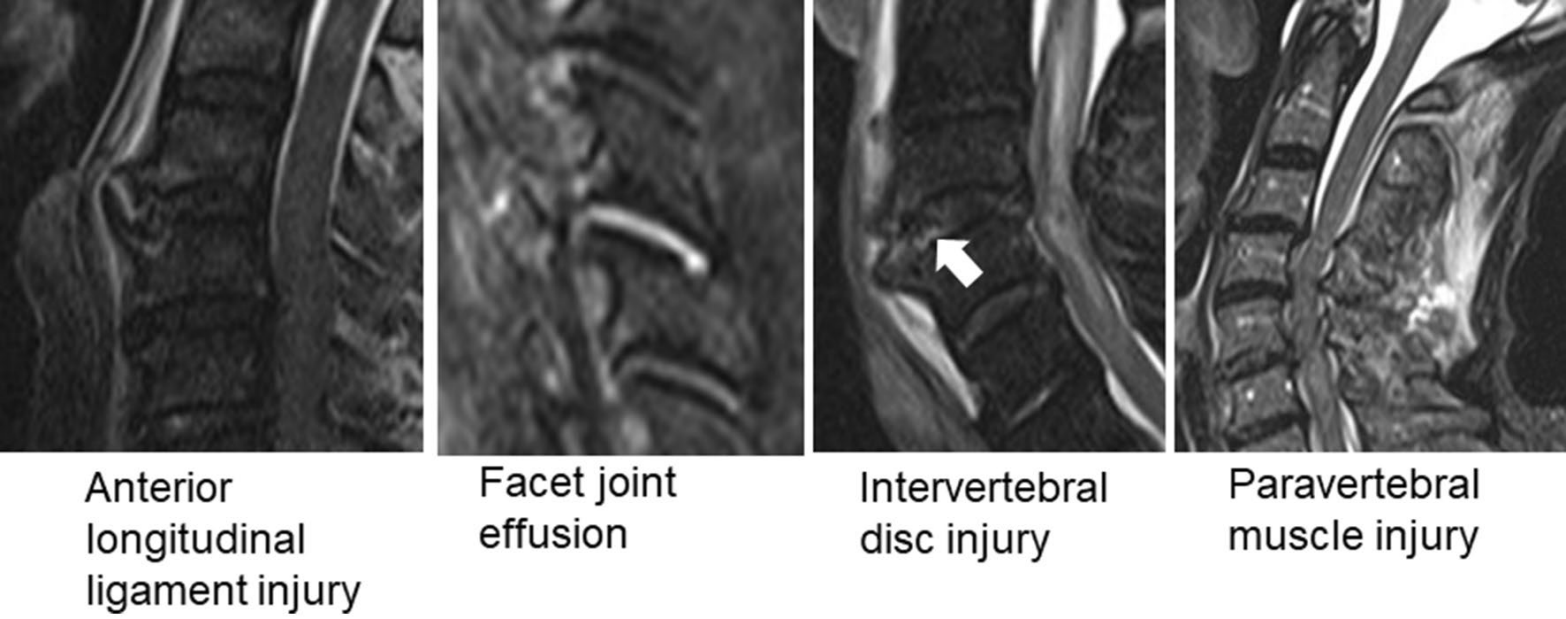
Assessment of cervical spine injury via magnetic resonance imaging (MRI, anterior longitudinal ligament injury, facet joint effusion, intervertebral disc injury (white arrow), and paravertebral muscle injury were assessed on T2-weighted MRI images of the cervical spine or short tau inversion recovery images.

ALL injury, facet joint effusion, intervertebral disc injury, and paravertebral muscle injury were assessed on T2-weighted MRI images of the cervical spine or short tau inversion recovery images (Fig. 1). Positive findings on MRI were evaluated independently by two expert spine surgeons, and the images of those who provided consent for the examination were analyzed.

### Statistical analysis

The MRI findings of cervical spine injury were assessed using the Fisher’s exact test in patients with segmental instability (group A) and those without (group B). To assess the risk factors of spinal instability on MRI, a logistic regression analysis was performed using instability confirmed on radiography as the dependent variable and MRI findings as the explanatory variables. A P value of < 0.05 was considered statistically significant. The Statistical Package for the Social Sciences software version 26 was used (IBM Corp., NY, the USA).

### Ethics approval and consent to participate

All procedures performed in studies involving human participants were in accordance with ethical standards, and this research was approved by the Ethics Committee of the Faculty of Medicine of Tottori University (Approval No. 19A041).

This study was conducted in accordance with the Declaration of Helsinki, and data confidentiality was ensured. We did not obtain the informed consent from the patients directly. We used opt-out to obtain consent for participation in this study. However, some patients died before the study. Hence, informed consent was not obtained. The Tottori University Ethics Committee has decided to exclude cases that have already died if there is a comment from the family that refuses consent due to opt-out. Actually, there were no cases waived by the families offer.

## Results

We treated 45 participants (30 men and 15 women) with spinal injury without vertebral fractures.

In total, 11 participants who could not undergo dynamic radiography (n = 4, unconsciousness caused by brain injury; n = 1 severe obesity; n = 1,mental retardation; and n = 5, multiple extremity fracture and multiple organ injury) were not included.

Finally, we investigated 34 participants (25 men and 9 women), with an average age of 65.0 years. Instability was confirmed on dynamic radiography in 11 patients (8 male and 3 female, with an average age of 64.6 years) in Group A and 23 patients (17 men and 6 women, with an average age of 65.3 years) in group B. Table 1 shows the demographic characteristics of the participants.

**Table1:** Patient demographics.

	Group A (n = 11)	Group B (n = 23)
Age (years)	63.4 ± 10.8	65.3 ± 16.3
Gender (male:female)	8:3	17:6
Treatment (conservative treatment:surgical treatment)	2:9	22:1
**ASIA Impairment Scale**		
E	2	8
D	5	4
C	3	7
B	0	4
A	1	0
**Injury level**		
C2/3	0	1
C3/4	2	6
C4/5	1	6
C5/6	5	9
C6/7	3	1

Values are mean ± standard deviation.

In most cases, the injury was at the middle and lower cervical spine. MRI revealed that there was a significantly higher proportion of patients with ALL injuries, intervertebral disc injuries, and bilateral facet joint effusions in group A than in group B (Table 2). A logistic regression analysis was performed using ALL injury, intervertebral disc injury, and bilateral facet joint effusion, which significantly differed between the two groups, as the explanatory variables, and instability on radiography as the dependent variable. Bilateral facet joint effusion was a significant risk factor (95% confidence interval: 1.31–84.7, odds ratio: 10.6, P = 0.03; Table 3).

**Table 2:** MRI findings.

	Group A (n = 11)	Group B (n = 23)	P value
Anterior longitudinal ligament injury	6/11	5/23	< 0.05
Paravertebral muscle injury	10/11	16/23	0.25
Intervertebral disc injury	5/11	2/23	< 0.05
Facet joint effusion (hemi lateral)	2/11	7/23	0.37
Facet joint effusion (bilateral)	7/11	7/23	< 0.05

**Table 3:** The association factor of segment instability with MRI findings.

MRI findings	Odds ratio	95% Confidence interval	P value
Anterior longitudinal ligament injury	2.12	0.15–30.5	0.58
Intervertebral disc injury	2.12	0.15–30.5	0.58
Facet joint effusion (bilateral)	10.6	1.31–84.7	0.03

**Figure 2:**
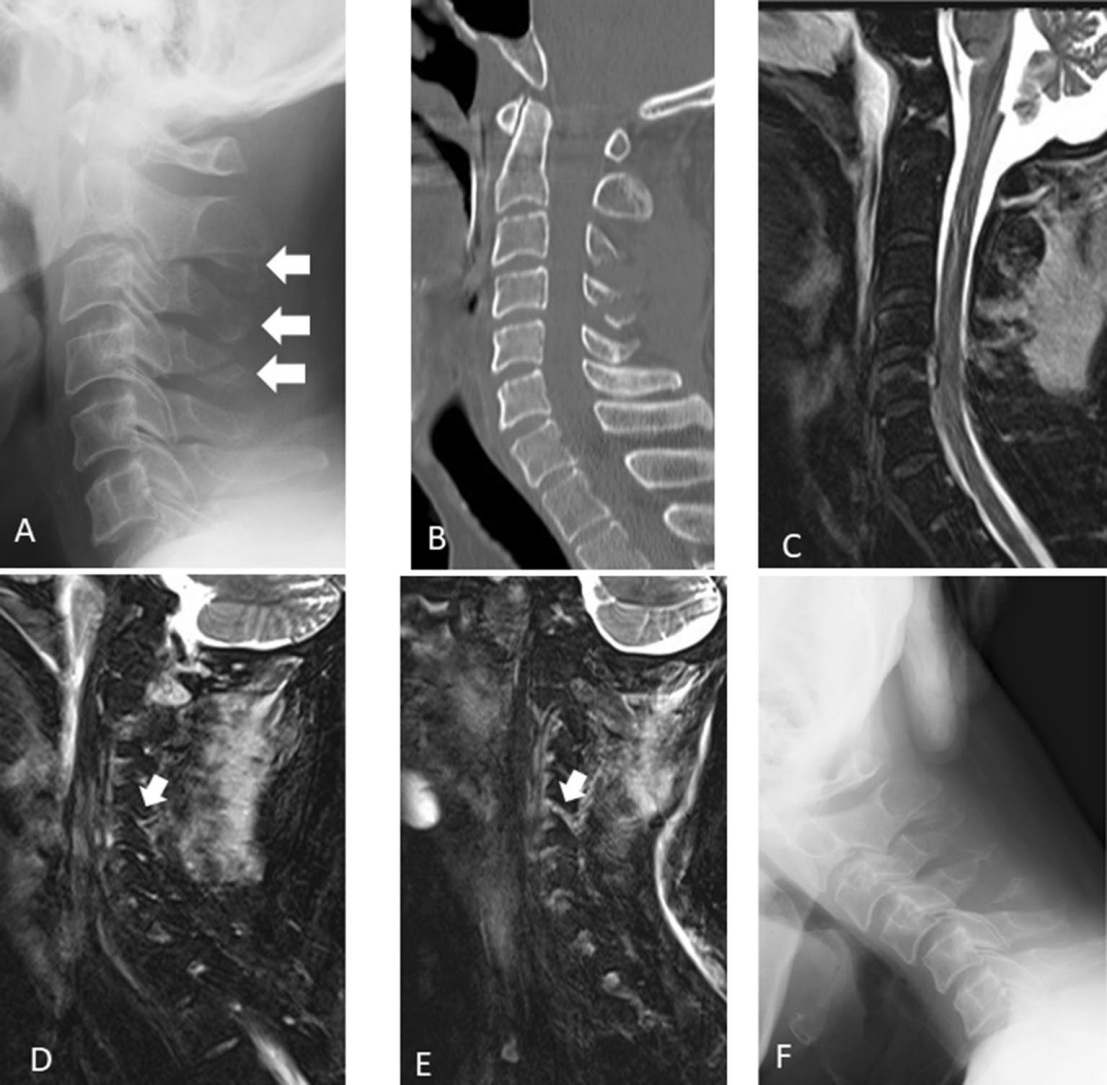
Assessment of cervical instability in a 56-year-old patient. (**A**,**B**) Lateral radiography image obtained at neutral position during the initial visit and mid-sagittal view of computed tomography scan. Radiography showed C2, 3, 4 spinous process fracture (white arrow). (**C**) Mid-sagittal view on magnetic resonance imaging and short tau inversion recovery image. (**D**,**E**) C4/5 facet effusion (white arrow). (**F**) Dynamic radiography showing segmental instability at C4/5.

Figure 2 depicts a typical case of segmental instability with facet effusion on dynamic radiography. The sensitivity and specificity of bilateral facet joint effusion indicating cervical segmental instability were 0.73 and 0.60, respectively.

## Discussion

Cervical segmental instability after cervical spine trauma is challenging to assess. In particular, according to the classification of Allen^7^, stage 4 distractive flexion injury is an extremely unstable condition accompanied by bilateral facet joint dislocation, in addition to damage in the supporting soft tissues. However, it may be overlooked if it has been spontaneously reduced. Thesleff et al^8^ reported the causes of inappropriate initial diagnosis among 2,041 cases, in which spinal injuries led to death. In this study, 206 (13.3%) cases of spinal injuries were coverlooked. However, the study was conducted at another facility, and it was not described in detail what type of cervical spine trauma had been delayed in the diagnosis. The diagnosis was overlooked because cervical spine trauma was not suspected during the initial assessment, and thorough imaging studies were not performed. In the current study, segmental instability likely occurred in cases of bilateral facet joint effusion. This finding is a good indicator of instability in cervical spine injury without evident dislocation or vertebral fracture. The assessment of cervical segmental instability via dynamic imaging is useful for assessing cervical spine instability that has been spontaneously reduced. Maeda et al. performed dynamic imaging of the cervical spine on more than 200 patients with cervical spine trauma on the day of injury. This is effective for diagnosing recoil distraction injuries and examination-associated exacerbation of spinal cord disorder^5^. Although dynamic imaging can be safely performed by an experienced spine surgeon, whether it can be immediately applied at all facilities has not been validated. In addition, this imaging method has some disadvantages, such as difficulty in assessing the lower cervical spine^9^ and undeniable risk of obtaining false positive and negative results^10^. Pollack et al. performed a retrospective study on the imaging findings of cervical spine injury in 818 patients at 21 facilities. In this study, dynamic imaging was performed on 86 (10.5%) patients, and the procedure might have caused spinal cord injury in 19 cases. Further, in four cases, cervical segmental instability was assessed via dynamic imaging. Thus, it was not considered useful, and it was applied as an auxiliary diagnostic approach to computed tomography scan and MRI^11^. Because this retrospective study used information from a specific database, the details of individual cases could not be identified. Moreover, it had some issues. That is, whether this imaging method had caused the exacerbation of spinal cord disorder was not confirmed, and the treatment course was unclear. However, it indicated a risk of developing new spinal cord disorder due to the techniques used by physicians during dynamic imaging.

Thus, if patients did not present with any complications after trauma, MRI is initially performed to identify the possible indicators of instability. Then, an experienced physician conducts dynamic imaging to confirm the findings. This is considered a safe and reliable method for detecting cervical segmental instability. Moreover, according to Maeda et al., intervertebral disc injury is an important MRI finding for assessing segmental instability. In the current study, the presence of facet joint effusion might indicate instability. Although MRI should be initially considered in the case of cervical spinal cord injury, findings that suggest instability are extremely significant for determining appropriate treatment strategies.

Furthermore, post-traumatic facet joint effusion can reflect hematoma in the facet joints after sustaining joint capsule injury caused by trauma. Facet joint capsule injury causes a high degree of segmental instability, which can be observed on dynamic imaging. The supportive property of facet joints is mainly responsible for the posterior cervical spine. However, in addition to hyperextension injury, hyperflexion injury may also occur. Therefore, instability caused by cervical spine trauma in all directions can be assessed. Moreover, the diagnosis of instability can be more effective when focusing on facet joint effusion, in addition to intervertebral disc injury. Nevertheless, this result does not deny the significance of dynamic imaging. In the current study, the sensitivity and specificity of facet joint effusion indicating cervical segmental instability were 0.73 and 0.60, respectively. This finding indicates an undeniable risk of obtaining false positive and negative results. Therefore, the use of combined dynamic radiography and MRI is essential. Although the number of cases in which instability was detected again on radiography was low, instability could be assessed using this imaging method in some cases.

The current study had several limitations. First, only a small number of participants were included. Second, because dynamic imaging was performed at the early stage of injury, patients are usually in pain. Thus, a thorough dynamic imaging could not be performed. Third, either an emergency physician or an orthopedic surgeon performs dynamic imaging. Hence, its process was inconsistent in some cases. Fourth, whether facet joint effusion is caused by existing segmental instability caused by degenerative diseases including rheumatoid arthritis and trauma is challenging to confirm. However, an evaluation of whether facet joint edema is attributed to trauma or whether degeneration is significant based on the presence or absence of soft tissue damage around the facet joint can be performed.

## Conclusions

Facet joint effusion might be caused by capsule injury attributed to trauma. Most participants presented with segmental instability accompanied by ALL, disc, and flavum injury and bilateral facet effusion. Thus, bilateral facet effusion and other types of soft tissue damage in the cervical spine can be considered as an indicator of spinal instability.
